# Effects of ginseng berry saponins on cardiorespiratory fitness in patients with SCAD: a randomized, double-blinded, placebo-controlled trial

**DOI:** 10.3389/fphar.2026.1724232

**Published:** 2026-01-22

**Authors:** Yaru Ge, Na Huan, Jinghui Sun, Tiantian Chao, Xiaohui Zhao, Chenglong Wang

**Affiliations:** 1 Beijing Shijitan Hospital affiliated with Capital Medical University, Beijing, China; 2 Xiyuan Hospital, China Academy of Chinese Medical Sciences, Beijing, China; 3 Jilin Ji’an Yisheng Company, Jilin, China

**Keywords:** cardiorespiratory fitness, randomized controlled trial, serum proteomics, stable coronary artery disease, Zhenyuan capsule

## Abstract

**Background:**

With the development of cardiac rehabilitation (CR), the advantages of combined Chinese and Western medicine cardiac rehabilitation for the treatment of stable coronary artery disease (SCAD) have become increasingly prominent.

**Purpose:**

This study was aimed to evaluate the effect of Zhenyuan capsule (a Chinese patented medicine consisting of ginseng berry saponins extracted from the mature berry of Panax Ginseng) on cardiorespiratory fitness (CRF) in patients with SCAD, and explore possible potential candidate molecule.

**Study Design and Methods:**

Using a randomized, double-blind, placebo-controlled trial design, 100 patients with SCAD were enrolled and randomly divided into the group taking Zhenyuan capsules (test group, n = 50) and the group taking placebo (control group, n = 50). In both groups, patients were treated with secondary prevention medication for CHD, with the addition of 2 capsules of Zhenyuan capsule 3 times a day for 12 weeks in the test group and 2 capsules of placebo 3 times a day for 12 weeks in the control group, with a follow-up of 1 month after the end of treatment. Subjects completed symptom-limited maximal cardiopulmonary exercise test (CPET) on a bicycle ergometer at 3 time points before enrollment, after 12 weeks of treatment, and after 1 month of follow-up. The main outcome was the increase in metabolic equivalent.

**Results:**

Both at anaerobic threshold (AT) level and at maximal level, the results of between-group comparisons showed that the test group was significantly better than that of the control group after treatment (AT level: 0.58 ± 0.89 Mets vs. 0.15 ± 1.06 Mets; maximal level: 0.69 ± 0.92 Mets vs. 0.19 ± 0.93 Mets) (*P* < 0.05). No serious adverse events occurred in patients in both groups. Serum proteomics studies suggested that insulin-like growth factor II (IGF2) was downregulated in a minority of responders, potentially related to cholesterol metabolism and the PI3K-Akt pathway.

**Conclusion:**

Zhenyuan capsule can significantly improve the CRF of patients with SCAD, and the downregulation of IGF2 observed in a minority of responders suggests IGF2 may be a potential candidate molecule of interest associated with cholesterol metabolism and the PI3K-Akt pathway.

**Clinical Trial Registration:**

https://www.chictr.org.cn/showproj.html?proj=53361, identifier ChiCTR2000032818.

## Graphical Abstract

**Figure:**
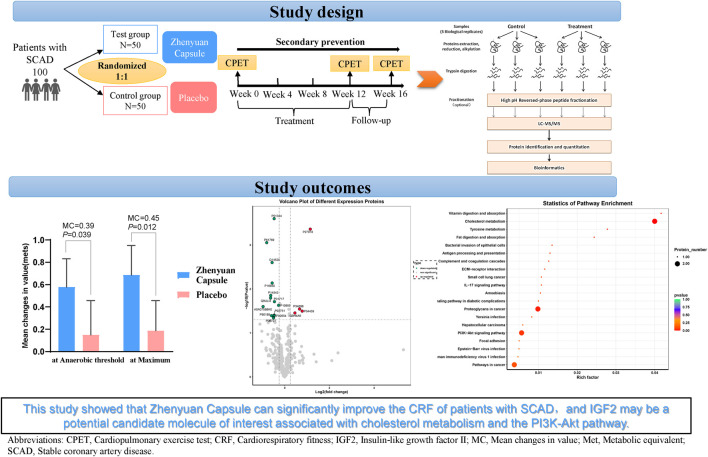

## 1 Introduction

Stable coronary artery disease (SCAD) is the main clinical type of coronary heart disease (CHD), which seriously jeopardizes human health (Roth et al., 2020). There are still many problems with SCAD, such as restenosis after intervention, increased cardiovascular events, and reduced quality of life (Boden et al., 2007; De Bruyne et al., 2012; Frye et al., 2009; Maron et al., 2020; Sedlis et al., 2015). Cardiorespiratory fitness (CRF) is the primary measurement component of cardiac rehabilitation (CR) and is a marker of generalized health (Corrà et al., 2010; Fletcher et al., 2013). Higher CRF improves clinical benefit of SCAD (Anderson et al., 2016; McMahon et al., 2017; Oldridge et al., 1988; Tutor et al., 2022). Although exercise therapy is a major component of CR, it is highly dependent on patient compliance, and its therapeutic effect is greatly reduced in patients with low compliance. As one of the “Five Major Prescriptions” for CR, pharmacologica prescriptions not only improve CRF but are also more readily accepted by patients.

Zhenyuan capsule is a capsule preparation developed from the saponin of ginseng fruit extract. Ginseng fruit and ginseng (heel) have the same main components, all of which are ginsenosides. Unlike the ginseng root, the saponins in ginseng fruit are primarily ginsenoside Re, whose content is significantly higher than that in the main ginseng root. Ginsenoside Re is one of the key saponin components in ginseng (Choi et al., 2012). With the help of high performance liquid chromatography (HPLC), ginsenoside Re can be well separated from other ginsenoside components, so that the content of ginsenoside Re in Zhenyuan capsule can be detected accurately (Xie et al., 2002). Zhenyuan Capsule has been approved for the treatment of CAD, angina pectoris and type 2 diabetes by the China Food and Drug Administration (drug approval No. Z22026091) for more than 20 years.

Previous study showed that Zhenyuan capsule could improve angina symptoms in CHD patients and have no significant adverse effects (Shi et al., 2019). However, it is unclear whether Zhenyuan capsule can increase CRF in SCAD patients. Therefore, this study was conducted to objectively evaluate the efficacy and safety of Zhenyuan capsule on the CRF of SCAD patients, and explore possible potential candidate molecule.

## 2 Materials and methods

### 2.1 Setting and participants

SCAD patients attending the cardiovascular department of Xiyuan Hospital, China Academy of Traditional Chinese Medicine (CATCM) from November 2020 to December 2021. Diagnostic criteria for Chinese medicine evidence refers to the diagnostic criteria for thoracic paralytic heartache (coronary heart disease myocardial infarction) in the Diagnostic Efficacy Criteria for Chinese Medicine Evidence formulated by the State Administration of Traditional Chinese Medicine (SATCM) in 2012,and the Guiding Principles for Clinical Research on New Traditional Chinese Medicines for the Treatment of Thoracic Paralysis (Angina Pectoris of Coronary Heart Disease) formulated by the Ministry of Health of the People’s Republic of China in 2002.

Western medical diagnostic criteria refers to the Chinese Guidelines for the Diagnosis and Treatment of Stable Coronary Heart Disease issued by the Chinese Society of Cardiovascular Disease of the Chinese Medical Association in 2018 (Section of Interventional Cardiology of Chinese Society of Cardiology, 2018) and the 2010 Chinese Consensus on the Management of Chronic Stable Coronary Heart Disease.

Inclusion and exclusion criteria are detailed in Supplementary Figure S1.

### 2.2 Randomization and interventions

Randomization was generated by a dedicated person using Stata software, a statistician who was not involved in this study. This study was double-blinded to investigators and subjects, and the codes were produced after the randomization operation.

Zhenyuan capsule 0.25g/capsule (Batch No. 200925)and Zhenyuan capsule simulant 0.25g/capsule were supplied by Jilin Ji’an Yisheng Pharmaceutical Co, and all experiments used the same batch of drugs. Each package was required to uniformly affix a label with a drug label number, which consisted of the drug name, indication, drug number, specification, storage, dosage, quantity, batch number, period of use, and the unit of supply of the drug, and was indicated to be a label for clinical research use only. The placebo appearance packaging, taste and odor were similar to that of Zhenyuan capsules. Neither the investigator nor the subjects were aware of the order of study treatment.

### 2.3 Study process

Using a randomized, double-blind, placebo-controlled clinical research trial design, 100 SCAD patients were enrolled according to the inclusion and exclusion criteria and randomly divided into the group taking Zhenyuan capsule (test group, n = 50) and the group taking placebo (control group, n = 50). Patients in both groups were treated with drugs for secondary prevention of CHD, and 2 capsules of Zhenyuan capsule were added to the test group, 3 times a day for 12 weeks; 2 capsules of placebo were added to the control group, 3 times a day for 12 weeks, and the patients were followed up for 1 month at the end of the treatment. Subjects underwent cardiopulmonary exercise test (CPET) at all 3 time points: before enrollment, after 12 weeks of treatment, and after 1 month of follow-up. The CPET system used in this study was provided by JAEGER’s MasterScreen CPX from Germany. The test was conducted on a bicycle dynamometer in accordance with the standards of the CPET laboratory at the University of California, Los Angeles Medical Center, and was a symptom-limited maximal CPET. For a detailed CPET plan, please refer to the Supplementary Material.

### 2.4 Ethical principles

The clinical trial was approved by the Ethics Committee of Xiyuan Hospital, China Academy of Traditional Chinese Medicine (2020XLA018-2), and registration was completed in Chinese Clinical Trial Registry (ChiCTR2000032818, Registered 11 May 2020, https://www.chictr.org.cn/showproj.html?proj=53361). All participants provided written informed consents before enrollment.

### 2.5 Research preparation: standardized description of zhenyuan capsules

Zhenyuan Capsules are capsules made from total saponins extracted from mature ginseng fruit. The product contains total saponins from ginseng fruit, calculated as ginsenoside Re, at 85%–115% of the labeled amount. Total ginseng fruit saponins are extracted from the mature fruits (pitted) of *Panax ginseng C.A.Mey.*, a plant of the Araliaceae family. Each batch of Zhenyuan Capsules is accompanied by a complete Finished Product Inspection Report (see Supplementary Figure S2), with testing conducted strictly according to the standards of the Chinese Pharmacopoeia. We provide a chemical fingerprint based on HPLC for monitoring batch-to-batch consistency (see Supplementary Figure S3) and quantitative determination of key active components (see Supplementary Table S1).

### 2.6 Outcomes

The main outcome is the increase in metabolic equivalent (Mets), and the secondary outcomes are peak oxygen uptake (VO_2_ peak), O_2_ pulse, respiratory exchange ratio (RER), 1min after exercise cessation 1min heart rate recovery (HRR1), 2min heart rate recovery (HRR2), duration of exercise. Safety indicators included vital signs, blood, urine and stool routines, liver and kidney function, electrocardiogram and adverse events. The standard operating procedure (SOP) for subject reception and the technology route are detailed in Supplementary Figures S4, S5. Full analysis set (FAS) and Per protocol set (PPS) were performed for efficacy indicators.

### 2.7 Statistical analysis

In this trial, the metabolic equivalents (ΔMETs) before and after treatment were used as the main efficacy index to estimate the sample coming size. Combined with the Based on relevant literature (Fan et al., 2021; Letnes et al., 2019) and prior data, we anticipate a between-group difference (δ) of 1.2 post-intervention and a pooled standard deviation (σ) of 2.03. With a two-sided α level of 0.05% and 80% test power, calculations using the formula for comparing two independent sample means indicate that 45 subjects per group (90 subjects total) are required without accounting for dropouts. To accommodate an estimated 10% dropout rate, the sample size per group was expanded to 50 subjects. Consequently, the study will recruit 100 subjects (50 per group).

Continuous variables are described by mean with standard deviation (SD), counts are described by the number of cases and the constitutive ratio [n (%)]. The two independent samples t-test or the nonparametric test were used; the Pearsonχ^2^ test or Fisher’s exact test was mainly used for the analysis of the count data; and the Wilcoxon rank-sum test was mainly used for the analysis of the hierarchical data.

For the analysis of primary and secondary continuous outcome measures, we employed linear mixed-effects models. Treatment strategy, age, sex, smoking history (yes/no), hyperlipidemia (yes/no), and diabetes (yes/no) were set as fixed effects, while patient identification number served as a random effect. All categorical covariates were included as factors in the model. Results are presented as mean values with 95% confidence intervals (95% CI). This model incorporated all available data from randomized subjects, addressing missing values due to follow-up loss through holographic maximum likelihood estimation. Under the assumption of random data loss, this method provides unbiased and efficient estimates, outperforming completion-only analysis or simple imputation techniques.

Samples for proteomics analysis were derived from a subset of subjects demonstrating marked improvement in clinical endpoints. This non-random, outcome-based selection aimed to identify the strongest signals but inevitably introduced confirmation bias. Consequently, all resulting omics data should be regarded as exploratory, hypothesis-generating preliminary findings that do not represent the overall population nor support causal inferences. Statistical tests were performed using a two-sided test of variance with a statistically significant *P* < 0.05. SPSS28.0 software was used for statistical analysis. Graphical presentation was performed using GraphPad Prism 8.

## 3 Results

### 3.1 Baseline characteristics

**FIGURE 1:**
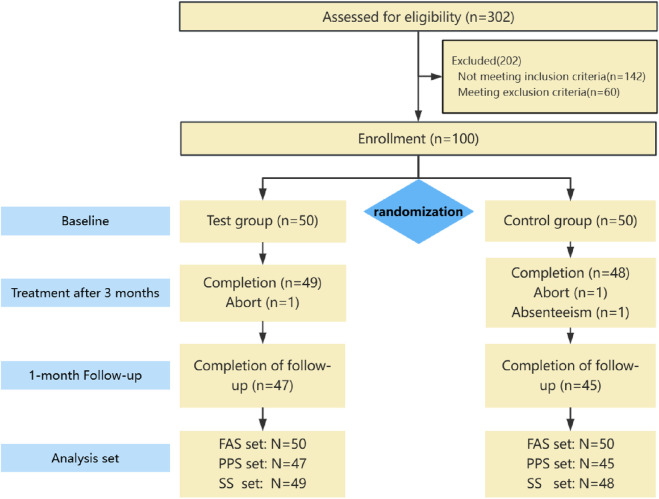
Flow chart of study. Abbreviations: FAS, Full analysis set; PPS, Per protocol set; SS, Safety set.

This study began with the inclusion of the first subject in November 2020 and ended with the last subject’s follow-up in December 2021, totaling 100 subjects. Subjects were discharged in 3 cases, and the overall rate of discharges was 3%. See Figure 1 for details.

Baseline characteristics of the participants were detailed in Table 1 and Supplementary Table S1. The mean age of all participants was 61.0 (SD, 8.4) years, with 76% of participants being male; mean BMI was 25.32 (SD, 2.33) kg/m^2^. The baseline characteristics were all comparable between the test and control groups.

**TABLE 1:** Baseline characteristics of study participants (FAS set).

Variables at baseline	All participants (n = 100)	Test group (n = 50)	Control group (n = 50)
Age (year), mean (*SD*)	61.0 (8.4)	62.2 (8.4)	59.9 (8.4)
Gender, n (%)			
Male	76 (76%)	35 (70%)	41 (82%)
Female	24 (24%)	15 (30%)	9 (18%)
Marital status, n (%)			
Unmarried	3 (3%)	1 (2%)	2 (4%)
Married	97 (97%)	49 (98%)	48 (96%)
BMI (kg/m^2^), mean (*SD*)	25.32 (2.33)	25.27 (2.31)	25.37 (2.38)
Education, n (%)			
<High school	20 (20%)	10 (20%)	10 (20%)
High school	30 (30%)	14 (28%)	15 (30%)
>High school	50 (50%)	26 (52%)	25 (50%)
SBP (mmHg), mean (*SD*)	124 (9)	124 (7)	125 (10)
DBP (mmHg), mean (*SD*)	76 (6)	75 (6)	77 (7)
HR (bpm), mean (*SD*)	68 (7)	67 (6)	69 (7)
History of hemodynamic reconstruction, n (%)			
No	36 (36%)	19 (38%)	17 (34%)
PCI	61 (61%)	29 (58%)	32 (64%)
CABG	3 (3%)	2 (4%)	1 (2%)
Risk factors, n (%)			
Smoking	58 (58%)	24 (48%)	34 (68%)
Hypertension	60 (60%)	30 (60%)	30 (60%)
Hyperlipidemia	85 (85%)	46 (92%)	39 (78%)
Diabetes	32 (32%)	20 (40%)	12 (24%)
TC (mmol/L), mean (*SD*)	3.69 (0.83)	3.62 (0.64)	3.65 (0.82)
LDL-C (mmol/L), mean (*SD*)	1.99 (0.64)	1.99 (0.61)	2.06 (0.72)
HDL-C (mmol/L, mean (*SD*)	1.11 (0.25)	1.11 (0.27)	1.09 (0.22)
TG (mmol/L), mean (*SD*)	1.43 (0.92)	1.47 (0.92)	1.42 (1.00)
Antiplatelet drug, n (%)	90 (90%)	45 (90%)	45 (90%)
Lipid-lowering drug, n (%)	93 (93%)	48 (96%)	45 (90%)
Beta-blocker, n (%)	55 (55%)	27 (54%)	28 (56%)
Nitrate, n (%)	21 (21%)	11 (22%)	10 (20%)
ACEI/ARB, n (%)	37 (37%)	20 (40%)	17 (34%)
CCB, n (%)	22 (22%)	12 (24%)	10 (20%)
Echocardiographic parameters			
LVEF,%	62 (62)	63 (64)	62 (63)
LVEDD,mm	46 (47)	46 (46)	47 (48)
CPET indicator, mean (*SD*)			
Mets at AT (mets)	3.24 (0.78)	3.23 (0.74)	3.25 (0.84)
Mets at max (mets	4.82 (0.90)	4.82 (0.94)	4.82 (0.86)
VO_2_ peak (mL/min)	1,216 (284)	1,193 (296)	1,239 (272)
O_2_ pulse, mL	10.84 (5.76)	11.55 (9.15)	10.13 (2.41)
RER	1.02 (0.13)	1.01 (0.14)	1.03 (0.12)
HRR1 (bpm)	17 (9)	16 (9)	18 (8)
HRR2 (bpm)	25 (10)	24 (10)	27 (10)
Duration of exercise (S)	523 (106)	525 (107)	522 (102)

Abbreviations: AT, Anaerobic threshold; Bmi, body mass index; CABG, Coronary artery bypass grafting; DAPT, Duplex antiplatelet therapy; DBP, Diastolic blood pressure; HDL-C, High density lipoprotein cholesterol; HR, Heart rate; HRR1, 1min Heart Rate Recovery; HRR2, 2min Heart Rate Recovery; LDL-C, Low density lipoprotein cholesterol; LVEDD, Left ventricular end-diastolic diameter; LVEF, Left ventricular ejection fraction; Met, Metabolic equivalent; PCI, Percutaneous coronary intervention; RER, Respiratory exchange rate; SBP, Systolic blood pressure; SCAPT, Single-combination antiplatelet therapy; TC, Total cholesterol; TG, Triglycerides; VO_2_, Oxygen uptake.

### 3.2 Effects of Zhenyuan caspule on CRF in SCAD patients

**FIGURE 2:**
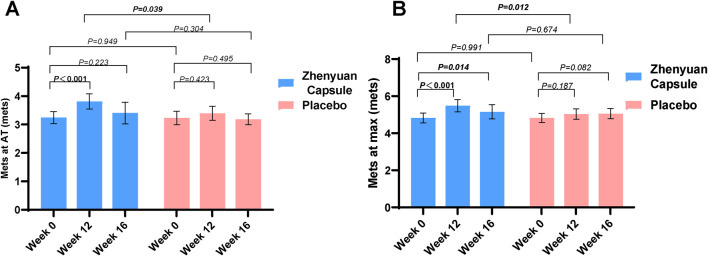
Metabolic equivalent changes during the study in both groups. **(A)** Shows Mets at AT and **(B)** shows Mets at maximum.

At the end of the 12-week treatment, in the FAS set (Figure 2), at the AT level, the test and control groups increased by 0.53mets and 0.13mets from baseline, respectively (95% CI: 0.29∼0.84, *P* < 0.001; 95% CI: −0.11∼0.51, *P* = 0.423), and the test group increased 0.39mets compared to the control group (95% CI: 0.03 to 0.81, *P* = 0.039); at the maximum level, the test and control groups increased by 0.63mets and 0.16mets from baseline, respectively (95% CI: 0.40 to 0.93, *P* < 0.001; 95% CI: −0.01 to 0.58, *P* = 0.187), the test group increased 0.45mets compared to the control group (95% CI: 0.12 to 1.89, *P* = 0.012) (see Figure 2 for details). VO_2_ peak in the test group increased by 81 mL/min (95% CI: 15∼178, *P* = 0.029), RER increased by 0.07 (95% CI: 0.02∼0.09, *P* < 0.001), HRR1 increased by 5 (95% CI: 1∼8, *P* = 0.012), HRR2 increased by 6 (95% CI:3∼12, *P* = 0.009). However, the differences between these metrics were not statistically significant when compared with placebo (Table 2; Supplementary Table S3 for details).

**TABLE 2:** Effects of Zhenyuan caspule and placebo on CRF in SCAD (FAS set).

Variables	Test group				Control group				Zhenyuan caspule vs. placebo	
	Baseline value	After treatment	Mean changes in value (95% CI)	*P* value	Baseline value	After treatment	Mean changes in value (95% CI)	*P* value	Mean changes in value (95% CI)	*P* value
Mets at AT (mets)	3.23 (0.74)	3.81 (0.93)	0.53 (0.29–0.84)	<0.001	3.25 (0.84)	3.40 (0.84)	0.13 (−0.11 to 0.51)	0.423	0.39 (0.04–0.91)	0.039
Mets at maximum (mets)	4.80 (0.94)	5.49 (1.15)	0.63 (0.40–0.93)	<0.001	4.84 (0.88)	5.03 (0.96)	0.16 (−0.01 to 0.58)	0.187	0.45 (0.12–1.89)	0.012
VO_2_ peak (mL/min)	1,185 (293)	1,274 (334)	81 (15–178)	0.029	1,235 (276)	1,240 (284)	5 (−72 to 89)	0.589	75 (−7 to 182)	0.388
O_2_ pulse (mL)	10.47 (2.23)	11.55 (9.15)	1.08 (-3.01–9.02)	0.879	10.15 (1.92)	10.13 (2.41)	0.03 (−1.27∼2.03)	0.683	1.05 (−1.42∼7.62)	0.902
RER	1.01 (0.14)	1.09 (0.11)	0.07 (0.02–0.09)	<0.001	1.04 (0.12)	1.10 (0.11)	0.06 (0.03–0.09)	0.003	0.01 (−0.12∼0.07)	0.702
HRR1 (bpm)	16 (9)	21 (10)	5 (1–8)	0.012	19 (8)	20 (13)	1 (−3.0 to 5.2)	0.423	4 (−1∼9)	0.201
HRR2 (bpm)	24 (10)	30 (12)	6 (3–12)	0.009	27 (10)	30 (12)	3 (−2.8 to 6.0)	0.208	3 (−1∼12)	0.209
Duration of exercise (S)	520 (134)	525 (107)	22 (−80 to 90)	0.505	534 (127)	522 (102)	−22 (−101 to 72)	0.389	44 (-180–113) (-191∼102)	0.398

Abbreviations: AT, Anaerobic threshold; CRF, cardiorespiratory fitness; HRR1, 1min Heart Rate Recovery; HRR2, 2min Heart Rate Recovery; RER, Respiratory exchange rate; SCAD, stable coronary artery disease; VO_2_, Oxygen uptake.

In this study, 92 subjects completed the 1-month follow-up at the end of the treatment period (47 in the test group and 45 in the control group.) At the end of the 1-month follow-up period, we performed CPET to assess the effect of Zhenyuan Capsules on the metabolic equivalents. A significant difference was found in the test group at the maximal level after 1 month of follow-up (4.79 ± 0.95 Mets at baseline vs. 5.16 ± 1.30 Mets at 1 month of follow-up; *P* = 0.014). The control group showed an increase in metabolic equivalents compared with pre-treatment, with no significant difference (baseline 4.86 ± 0.90 Mets vs. 5.06 ± 0.92 Mets at 1 month follow-up; *P* = 0.082) (Figure 2; Supplementary Table S4 for details).

### 3.3 Subgroup analysis

**FIGURE 3:**
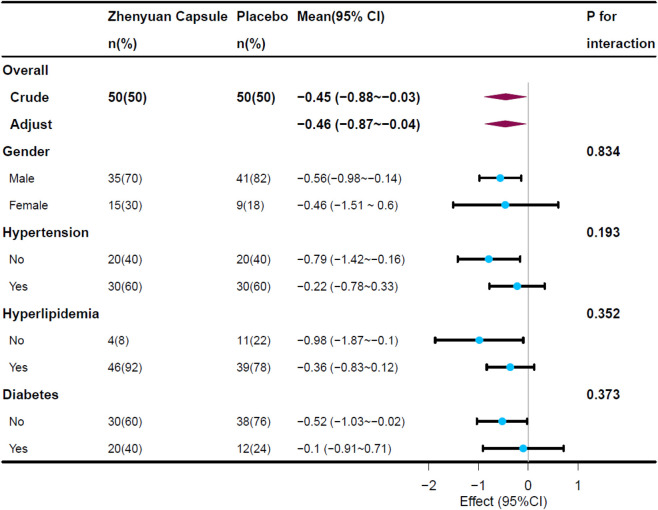
Changes of Mets at maximum across the subgroups.

In the subgroup analysis, Zhenyuan capsule increased CRF by 0.56mets (95% CI: 0.89–0.14, *P* = 0.011) in female patients compared to placebo. In patients without hypertension, hyperlipidemia, and diabetes mellitus, CRF was significantly increased with the use of Zhenyuan capsule (Figure 3; Supplementary Table S5 for detail).

### 3.4 Safety analysis

During the 12-week treatment period, there were no serious adverse events in either the test or control groups (Supplementary Table S6 for details). In addition, our analysis of pre- and post-treatment inter- and intra-group comparisons of safety metrics in the test and control groups revealed that the measured safety metrics were within normal count ranges, both pre- and post-treatment (Supplementary Tables S7 and S8 for details).

### 3.5 Mechanistic exploration based on proteomics

At the end of the clinical trial, 8 patients whose CRF was significantly improved after 3 months of treatment with Zhenyuan capsule were selected, and Label-free proteomics method was used to identify the differential protein expression profiles of patients before and after the treatment with Zhenyuan capsule, and the differential proteins obtained from the identification were mined and analyzed by bioinformatics analysis method, in order to explore the possible role of Zhenyuan capsule in improving CRF. The different proteins identified were mined and analyzed by bioinformatics analysis to explore potential biomarkers for the enhancement of Zhenyuan capsule in improving CRF. This non-random, outcome-based selection method aims to identify the strongest signals but inevitably introduces confirmation bias.

**FIGURE 4:**
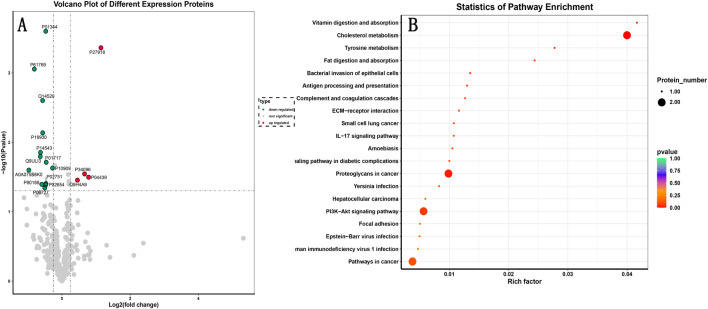
Proteomics-based mechanism of CRF enhancement by Zhenyuan capsule. **(A)** Volcano plot of differential protein expression. P01344 indicates significant downregulation of IGF2 (*P* < 0.001), P27918 indicates significant upregulation of CFP (*P* < 0.001). **(B)** Bubble plot of GO enrichment analysis of differential proteins.

The results showed that insulin-like growth factor II (IGF2) was significantly downregulated and properdin was significantly upregulated after treatment. KEGG analysis of the different proteins identified yielded a total of 40 pathways involving cholesterol metabolism, antigen processing and presentation, PI3K/Akt signaling pathway, vitamin digestion and absorption, fat digestion and absorption, complement and coagulation pathway, ECM-receptor interaction, IL-17 signaling pathway, etc. (Figure 4 for details).

## 4 Discusssion

The results of this study used the increase in metabolic equivalent as the main efficacy indicator (FAS set). The increase in metabolic equivalent at the AT level was 0.53 ± 0.92 Mets in the test group and 0.13 ± 0.98Mets in the control group; the increase in metabolic equivalent at the maximum level was 0.63 ± 1.02Mets in the test group and 0.16 ± 1.21Mets in the control group; comparison of both the AT level and the maximal level showed that the test group was significantly higher than the control group (*P* < 0.05). In addition, there was a significant difference in metabolic equivalents before and after treatment in the test group (*P* < 0.05). It indicates that Zhenyuan capsule can significantly improve the metabolic equivalents at AT and maximum. Sensitivity analysis suggested that the results of CRF-related indexes in the FAS set and the PPS set remained consistent, indicating that the shedding cases had little effect on the results of this study.

This study observed improvements in METs ranging from 0.39 to 0.45 at AT level and maximum level after treatment in the test group. We note that this value falls below the commonly cited threshold for ‘strong clinical relevance’ (typically ≥1.0 Met) proposed in some guidelines and reviews. Kodama et al. published the first meta-analysis on the predictive validity of CRF for health outcomes, finding that a 1-Met (3.5 mL/kg/min) increase in CRF was associated with a 13% reduction in all-cause mortality risk and a 15% reduction in CVD event risk (Kodama et al., 2009). This study helped establish 1-Met as the meaningful clinical difference in exercise testing. However, interpretation of this effect requires consideration of the following context: (1) patient population characteristics: our participants were SCAD receiving standard drug therapy, whose physiological reserve and potential for improvement may have been partially limited. Any statistically significant improvement in this group holds positive value; (b) nature and duration of intervention: Zhenyuan Capsule differs in intensity and mechanism from high-intensity exercise training or certain potent medications. The consistent improvement trend observed over a relatively short intervention period suggests positive physiological effects. Studies also indicate that even a modest improvement of 0.5 Mets may significantly enhance health outcomes (Bonafiglia et al., 2021; Lang et al., 2024). This study demonstrates that the Zhenyuan Capsules exhibit good tolerability with no reports of serious adverse events. Therefore, the clinical net benefit is positive, as it achieves statistically significant functional improvement with minimal additional risk.

The ventilatory anaerobic threshold (VAT), also known as the anaerobic threshold (AT), arises when the metabolic demands of exercise begin to exceed the delivery of oxygen to the working muscles, and blood lactate levels and lactate/pyruvate begin to rise, and is an index used to estimate exercise capacity (Milani et al., 2004; Millsaps and Ralph, 1987; Neuberg, 1988). As far as exercise prescription is concerned, VO_2_ peak and AT are the gold standard for developing an aerobic exercise prescription. AT can help differentiate between cardiac and noncardiac (lung or skeletal muscle) causes of exercise limitation, and patients who fatigue before reaching AT may have noncardiac problems (Brooks, 1985; Jennings and Esler, 1990). However, it has also been shown that patients with mitral stenosis stop exercising before reaching AT (Albouaini et al., 2007). In our study (FAS set), the metabolic equivalents increased from 3.23 ± 0.74 Mets to 3.81 ± 0.93 Mets in the test group and from 3.25 ± 0.83 Mets to 3.40 ± 0.84 Mets in the control group, which was significantly better than the control group after the treatment (*P* < 0.05), and there was a significant difference between before and after the treatment in the test group (*P* < 0.05).

The respiratory exchange ratio (RER) represents the metabolic exchange of gases in body tissues and is partially dependent on the main fuels of cellular metabolism (carbohydrates and fats) (Gibbons et al., 2002). Since RER levels are directly related to the accumulation of muscle lactate, they can be used as an objective means of quantifying effort during exercise (Milani et al., 2006). In our study, the median RER level increased from 1.01 to 1.09 in the test group and from 1.04 to 1.10 in the control group, with a statistically significant difference in the within-group comparison between the two groups before and after treatment (*P* < 0.05), but no statistically significant difference in the between-group comparison (*P* > 0.05).

Overall, compared to pre-treatment levels, metabolic equivalents and RER increased significantly while total exercise duration showed no marked increase, indicating that this treatment enabled patients to tolerate higher exercise intensity. Combined with the fact that there was no significant change in maximal heart rate before and after the treatment, this suggests that the patients were able to withstand higher-power exercise loads with the same heart rate, which is a manifestation of the improvement in the patients’ CRF.

The results of our study showed (FAS set) an increase in HRR1 from 16 (9) to 21 (10) bpm and HRR2 from 24 (10) to 30 (12) bpm in the patients of the test group after the treatment, with significant differences between the before and after treatments (*P* < 0.05). Heart rate recovery (HRR) refers to the rate at which heart rate decreases within minutes after exercise cessation. It reflects the dynamic equilibrium and synergistic interaction between parasympathetic reactivation and sympathetic inhibition (Borresen and Lambert, 2008; Cole et al., 1999; Coote, 2010; Peçanha et al., 2014). In contrast, heart rate after cessation of exercise and during the first 30 s of recovery is predominantly influenced by reactivation of the parasympathetic nervous system (Imai et al., 1994). Epidemiologic studies suggest that HRR may be a potential prognostic marker for predicting health outcomes, including cardiovascular disease (Ho et al., 2010; Mora et al., 2003; Morshedi-Meibodi et al., 2002; Newman et al., 2006; Park et al., 2015). In addition, studies have shown that HRR helps predict the risk of all-cause mortality (Aktas et al., 2004; Carnethon et al., 2012; Johnson and Goldberger, 2012; Savonen et al., 2011; Shetler et al., 2001; Wändell et al., 2010). A study from a meta-analysis showed that compared with references in the general population, a decrease in HRR was associated with an increased risk of cardiovascular events and all-cause mortality, and that there was no significant difference between HRR2 and HRR1 in predicting the risk of all-cause mortality (Qiu et al., 2017).

Proteomics studies revealed significant downregulation of IGF2 following treatment, involving cholesterol metabolism and the PI3K-Akt signaling pathway. However, due to limitations in study design and data acquisition, we were unable to perform correlation analyses between specific proteins (such as IGF2) and efficacy indicators. Therefore, we believe that the downregulation of IGF2 observed in a small number of respondents suggests it may be a potential candidate molecule worthy of attention, associated with cholesterol metabolism and the PI3K-Akt pathway. The study of the insulin-like growth factor family (IGFs) is a hot topic in cell biology today and is receiving increasing attention. IGF2 was the first confirmed endogenous imprinted gene. Loss of imprinting of the IGF2 gene has been found in a variety of tumors, including breast, lung and colon cancers (Bergman et al., 2013). Results of large-scale genetic studies have shown that the IGF2 gene region is associated with metabolic syndrome, type II diabetes mellitus and CAD (Rodríguez et al., 2004). The researchers found that circulating IGF2 did not affect atheromatous plaque formation, but increasing localized IGF2 levels in smooth muscle cells resulted in focal intimal thickening. Disruption of the IGF2 gene in ApoE^−/−^ mice was able to reduce the area of atheromatous plaque lesion damage and markedly reduced intimal thickening, suggesting that increased IGF2 expression may be an important mechanism in the pathogenesis of atherosclerosis (Zaina et al., 2002). These findings collectively provide a potential hypothesis for future research, suggesting that the vibration-generating capsule may exert its effects by influencing the aforementioned biological networks.

Future research should focus on extending intervention cycles, optimizing dosages, or exploring combination with other rehabilitation methods to determine whether more clinically meaningful improvements (e.g., ≥1.0 Met) can be achieved. Concurrently, these findings require validation in larger sample sizes, and efforts should be made to identify patient subgroups most likely to benefit from this intervention. In prospective designs, omics analyses should be conducted on all or randomly selected subjects to impartially validate potential signals observed in this study. Finally, gain-of-function and loss-of-function experiments in cellular and animal models are essential to establish the causal role of key molecules (e.g., IGF2) in mediating therapeutic effects.

## 5 Strengths and limitations

The study has several limitations. Firstly, this clinical trial study was set up as a single center with a relatively small sample size, resulting in significant differences in only within-group comparisons for some of the secondary efficacy indicators. Secondly, there was no long-term follow-up of patients for 6 months or even 1 year in order to allow a larger number of patients to be entered into the PPS set. Finally, the mechanistic exploration in this study has clear limitations:

1. Proteomic analysis was performed only in a small number of clinical responders, posing a risk of confirmation bias in sample selection. Therefore, the identified differentially expressed proteins and pathways cannot be generalized.
2. Due to study design limitations, we could not validate the direct correlation between changes in key molecules (such as IGF2) and individual clinical improvement, nor did we have functional experiments to support their causal role. Therefore, all findings in this section must be strictly regarded as preliminary clues and sources of hypotheses for future research, rather than conclusive evidence. Although some baseline characteristics were imbalanced after randomization in this study, we adjusted for these variables in the primary analysis using statistical models, and the robustness of the results was supported by subgroup analyses.

We highlight the following strengths. The study found that Zhenyuan capsule can significantly improve the CRF of SCAD, and the ability of HRR; the patients’ acceptance of Zhenyuan capsule is high.

## 6 Conclusion

In our study, through randomized, double-blind, placebo-controlled clinical research, Zhenyuan capsule can significantly improve the CRF of patients with SCAD, with high safety and good short-term prognosis, and IGF2 may be a potential candidate molecule associated with cholesterol metabolism and the PI3K-Akt pathway.

## Data Availability

The datasets presented in this study can be found in online repositories. The names of the repository/repositories and accession number(s) can be found in the article/Supplementary Material.

## References

[B1] Aktas M. K. Ozduran V. Pothier C. E. Lang R. Lauer M. S. (2004). Global risk scores and exercise testing for predicting all-cause mortality in a preventive medicine program. JAMA 292 (12), 1462–1468. 10.1001/jama.292.12.1462 15383517

[B2] Albouaini K. Egred M. Alahmar A. Wright D. J. (2007). Cardiopulmonary exercise testing and its application. Heart British Card. Soc. 93 (10), 1285–1292. 10.1136/hrt.2007.121558 17890705 PMC2000933

[B3] Anderson L. Oldridge N. Thompson D. R. Zwisler A.-D. Rees K. Martin N. (2016). Exercise-based cardiac rehabilitation for coronary heart disease: cochrane systematic review and meta-analysis. J. Am. Coll. Cardiol. 67 (1), 1–12. 10.1016/j.jacc.2015.10.044 26764059

[B4] Bergman D. Halje M. Nordin M. Engström W. (2013). Insulin-like growth factor 2 in development and disease: a mini-review. Gerontology 59 (3), 240–249. 10.1159/000343995 23257688

[B5] Boden W. E. O'Rourke R. A. Teo K. K. Hartigan P. M. Maron D. J. Kostuk W. J. (2007). Optimal medical therapy with or without PCI for stable coronary disease. N. Engl. J. Med. 356 (15), 1503–1516. 10.1056/NEJMoa070829 17387127

[B6] Bonafiglia J. T. Preobrazenski N. Islam H. Walsh J. J. Ross R. Johannsen N. M. (2021). Exploring differences in cardiorespiratory fitness response rates across varying doses of exercise training: a retrospective analysis of eight randomized controlled trials. Sports Med. 51 (8), 1785–1797. 10.1007/s40279-021-01442-9 33704698

[B7] Borresen J. Lambert M. I. (2008). Autonomic control of heart rate during and after exercise: measurements and implications for monitoring training status. Sports Med. Auckl. N.Z. 38 (8), 633–646. 10.2165/00007256-200838080-00002 18620464

[B8] Brooks G. A. (1985). Anaerobic threshold: review of the concept and directions for future research. Med. and Sci. Sports and Exerc. 17 (1), 22–34. 3884959

[B9] Carnethon M. R. Sternfeld B. Liu K. Jacobs D. R. Schreiner P. J. Williams O. D. (2012). Correlates of heart rate recovery over 20 years in a healthy population sample. Med. Sci. Sports Exerc. 44 (2), 273–279. 10.1249/MSS.0b013e31822cb190 21796053 PMC3838873

[B10] Choi S. Y. Cho C.-W. Lee Y. Kim S. S. Lee S. H. Kim K.-T. (2012). Comparison of ginsenoside and phenolic ingredient contents in hydroponically-cultivated ginseng leaves, fruits, and roots. J. Ginseng Res. 36 (4), 425–429. 10.5142/jgr.2012.36.4.425 23717146 PMC3659603

[B11] Cole C. R. Blackstone E. H. Pashkow F. J. Snader C. E. Lauer M. S. (1999). Heart-rate recovery immediately after exercise as a predictor of mortality. N. Engl. J. Med. 341 (18), 1351–1357. 10.1056/NEJM199910283411804 10536127

[B12] Coote J. H. (2010). Recovery of heart rate following intense dynamic exercise. Exp. Physiol. 95 (3), 431–440. 10.1113/expphysiol.2009.047548 19837772

[B13] Corrà U. Piepoli M. F. Carré F. Heuschmann P. Hoffmann U. Verschuren M. (2010). Secondary prevention through cardiac rehabilitation: physical activity counselling and exercise training: key components of the position paper from the cardiac rehabilitation section of the european association of cardiovascular prevention and rehabilitation. Eur. Heart J. 31 (16), 1967–1974. 10.1093/eurheartj/ehq236 20643803

[B14] De Bruyne B. Pijls N. H. J. Kalesan B. Barbato E. Tonino P. A. L. Piroth Z. (2012). Fractional flow reserve-guided PCI versus medical therapy in stable coronary disease. N. Engl. J. Med. 367 (11), 991–1001. 10.1056/NEJMoa1205361 22924638

[B15] Fan Y. Yu M. Li J. Zhang H. Liu Q. Zhao L. (2021). Efficacy and safety of resistance training for coronary heart disease rehabilitation: a systematic review of randomized controlled trials. Front. Cardiovasc Med. 8, 754794. 10.3389/fcvm.2021.754794 34805309 PMC8602574

[B16] Fletcher G. F. Ades P. A. Kligfield P. Arena R. Balady G. J. Bittner V. A. (2013). Exercise standards for testing and training: a scientific statement from the American heart association. Circulation 128 (8), 873–934. 10.1161/CIR.0b013e31829b5b44 23877260

[B17] Frye R. L. August P. Brooks M. M. Hardison R. M. Kelsey S. F. MacGregor J. M. (2009). A randomized trial of therapies for type 2 diabetes and coronary artery disease. N. Engl. J. Med. 360 (24), 2503–2515. 10.1056/NEJMoa0805796 19502645 PMC2863990

[B18] Gibbons R. J. Balady G. J. Bricker J. T. Chaitman B. R. Fletcher G. F. Froelicher V. F. (2002). ACC/AHA 2002 guideline update for exercise testing: summary article. A report of the American college of cardiology/american heart association task force on practice guidelines (committee to update the 1997 exercise testing guidelines). J. Am. Coll. Cardiol. 40 (8), 1531–1540. 10.1016/s0735-1097(02)02164-2 12392846

[B19] Ho J. S. Fitzgerald S. J. Barlow C. E. Cannaday J. J. Kohl H. W. Haskell W. L. (2010). Risk of mortality increases with increasing number of abnormal non-ST parameters recorded during exercise testing. Eur. J. Cardiovasc. Prev. Rehabilitation 17 (4), 462–468. 10.1097/HJR.0b013e328336a10d 20084008

[B20] Imai K. Sato H. Hori M. Kusuoka H. Ozaki H. Yokoyama H. (1994). Vagally mediated heart rate recovery after exercise is accelerated in athletes but blunted in patients with chronic heart failure. J. Am. Coll. Cardiol. 24 (6), 1529–1535. 10.1016/0735-1097(94)90150-3 7930286

[B21] Jennings G. L. Esler M. D. (1990). Circulatory regulation at rest and exercise and the functional assessment of patients with congestive heart failure. Circulation 81 (Suppl. l), 5–13. 2403869

[B22] Johnson N. P. Goldberger J. J. (2012). Prognostic value of late heart rate recovery after treadmill exercise. Am. J. Cardiol. 110 (1), 45–49. 10.1016/j.amjcard.2012.02.046 22463837

[B23] Kodama S. Saito K. Tanaka S. Maki M. Yachi Y. Asumi M. (2009). Cardiorespiratory fitness as a quantitative predictor of all-cause mortality and cardiovascular events in healthy men and women: a meta-analysis. JAMA 301 (19), 2024–2035. 10.1001/jama.2009.681 19454641

[B24] Lang J. A.-O. X. Prince S. A.-O. Merucci K. Cadenas-Sanchez C. A.-O. Chaput J. A.-O. Fraser B. A.-O. (2024). Cardiorespiratory fitness is a strong and consistent predictor of morbidity and mortality among adults: an overview of meta-analyses representing over 20.9 million observations from 199 unique cohort studies. Br. J. Sports Med. 58 (10), 556–566. 10.1136/bjsports-2023-107849 38599681 PMC11103301

[B25] Letnes J. M. Dalen H. Vesterbekkmo E. K. Wisløff U. Nes B. M. (2019). Peak oxygen uptake and incident coronary heart disease in a healthy population: the HUNT fitness study. Eur. Heart J. 40 (20), 1633–1639. 10.1093/eurheartj/ehy708 30496487

[B26] Maron D. J. Hochman J. S. Reynolds H. R. Bangalore S. O'Brien S. M. Boden W. E. (2020). Initial invasive or conservative strategy for stable coronary disease. N. Engl. J. Med. 382 (15), 1395–1407. 10.1056/NEJMoa1915922 32227755 PMC7263833

[B27] McMahon S. R. Ades P. A. Thompson P. D. (2017). The role of cardiac rehabilitation in patients with heart disease. Trends Cardiovasc. Med. 27 (6), 420–425. 10.1016/j.tcm.2017.02.005 28318815 PMC5643011

[B28] Milani R. V. Lavie C. J. Mehra M. R. (2004). Cardiopulmonary exercise testing: how do we differentiate the cause of dyspnea? Circulation 110 (4), e27–e31. 10.1161/01.CIR.0000136811.45524.2F 15277333

[B29] Milani R. V. Lavie C. J. Mehra M. R. Ventura H. O. (2006). Understanding the basics of cardiopulmonary exercise testing. Mayo Clin. Proc. 81 (12), 1603–1611. 10.4065/81.12.1603 17165639

[B30] Millsaps R. D. Ralph D. (1987). Principles of exercise testing and interpretation. Chest 92 (4), 30. 10.1016/s0012-3692(16)31282-x

[B31] Mora S. Redberg R. F. Cui Y. Whiteman M. K. Flaws J. A. Sharrett A. R. (2003). Ability of exercise testing to predict cardiovascular and all-cause death in asymptomatic women: a 20-year follow-up of the lipid research clinics prevalence study. JAMA 290 (12), 1600–1607. 10.1001/jama.290.12.1600 14506119

[B32] Morshedi-Meibodi A. Larson M. G. Levy D. O'Donnell C. J. Vasan R. S. (2002). Heart rate recovery after treadmill exercise testing and risk of cardiovascular disease events (the framingham heart study). Am. J. Cardiol. 90 (8), 848–852. 10.1016/s0002-9149(02)02706-6 12372572

[B33] Neuberg G. W. Friedman S. H. Weiss M. B. Herman M. V. (1988). Cardiopulmonary exercise testing. The clinical value of gas exchange data. Archives Intern. Med. 148 (10), 2221–2226. 10.1001/archinte.148.10.2221 3140752

[B34] Newman A. B. Simonsick E. M. Naydeck B. L. Boudreau R. M. Kritchevsky S. B. Nevitt M. C. (2006). Association of long-distance corridor walk performance with mortality, cardiovascular disease, mobility limitation, and disability. JAMA 295 (17), 2018–2026. 10.1001/jama.295.17.2018 16670410

[B35] Oldridge N. B. Guyatt G. H. Fischer M. E. Rimm A. A. (1988). Cardiac rehabilitation after myocardial infarction. Combined experience of randomized clinical trials. JAMA 260 (7), 945–950. 10.1001/jama.1988.03410070073031 3398199

[B36] Park J.-I. Shin S.-Y. Park S. K. Barrett-Connor E. (2015). Usefulness of the integrated scoring model of treadmill tests to predict myocardial ischemia and silent myocardial ischemia in community-dwelling adults (from the Rancho Bernardo study). Am. J. Cardiol. 115 (8), 1049–1055. 10.1016/j.amjcard.2015.01.536 25728643 PMC4380803

[B37] Peçanha T. Silva-Júnior N. D. Forjaz C. L. d. M. (2014). Heart rate recovery: autonomic determinants, methods of assessment and association with mortality and cardiovascular diseases. Clin. Physiology Funct. Imaging 34 (5), 327–339. 10.1111/cpf.12102 24237859

[B38] Qiu S. Cai X. Sun Z. Li L. Zuegel M. Steinacker J. M. (2017). Heart rate recovery and risk of cardiovascular events and all-cause mortality: a meta-analysis of prospective cohort studies. J. Am. Heart Assoc. 6 (5), e005505. 10.1161/JAHA.117.005505 28487388 PMC5524096

[B39] Rodríguez S. Gaunt T. R. O'Dell S. D. Chen X.-H. Gu D. Hawe E. (2004). Haplotypic analyses of the IGF2-INS-TH gene cluster in relation to cardiovascular risk traits. Hum. Mol. Genet. 13 (7), 715–725. 10.1093/hmg/ddh070 14749349

[B40] Roth G. A. Mensah G. A. Johnson C. O. Addolorato G. Ammirati E. Baddour L. M. (2020). Global burden of cardiovascular diseases and risk factors, 1990-2019: update from the GBD 2019 study. J. Am. Coll. Cardiol. 76 (25), 2982–3021. 10.1016/j.jacc.2020.11.010 33309175 PMC7755038

[B41] Savonen K. P. Kiviniemi V. Laaksonen D. E. Lakka T. A. Laukkanen J. A. Tuomainen T. P. (2011). Two-minute heart rate recovery after cycle ergometer exercise and all-cause mortality in middle-aged men. J. Intern. Med. 270 (6), 589–596. 10.1111/j.1365-2796.2011.02434.x 21801244

[B42] Section of Interventional Cardiology of Chinese Society of CardiologySection of Atherosclerosis and Coronary Artery Disease of Chinese Society of CardiologySpecialty Committee on Prevention and Treatment of Thrombosis of Chinese College of Cardiovascular Physicians (2018). Guideline on the diagnosis and treatment of stable coronary artery disease. Zhonghua Xin Xue Guan Bing Za Zhi 46 (9), 680–694. 10.3760/cma.j.issn.0253-3758.2018.09.004 30293374

[B43] Sedlis S. P. Hartigan P. M. Teo K. K. Maron D. J. Spertus J. A. Mancini G. B. J. (2015). Effect of PCI on long-term survival in patients with stable ischemic heart disease. N. Engl. J. Med. 373 (20), 1937–1946. 10.1056/NEJMoa1505532 26559572 PMC5656049

[B44] Shetler K. Marcus R. Froelicher V. F. Vora S. Kalisetti D. Prakash M. (2001). Heart rate recovery: validation and methodologic issues. J. Am. Coll. Cardiol. 38 (7), 1980–1987. 10.1016/s0735-1097(01)01652-7 11738304

[B45] Shi Z.-Y. Zeng J.-Z. Wong A. S. T. (2019). Chemical structures and pharmacological profiles of ginseng saponins. Mol. Basel, Switz. 24 (13), 2443. 10.3390/molecules24132443 31277214 PMC6651355

[B46] Tutor A. Lavie C. J. Kachur S. Dinshaw H. Milani R. V. (2022). Impact of cardiorespiratory fitness on outcomes in cardiac rehabilitation. Prog. Cardiovasc. Dis. 70, 2–7. 10.1016/j.pcad.2021.11.001 34780726

[B47] Wändell P. E. Carlsson A. C. Theobald H. (2010). Effect of heart-rate recovery on long-term mortality among men and women. Int. J. Cardiol. 144 (2), 276–279. 10.1016/j.ijcard.2009.01.053 19232445

[B48] Xie J.-T. Aung H. H. Wu J. A. Attel A. S. Yuan C.-S. (2002). Effects of American ginseng berry extract on blood glucose levels in ob/ob mice. Am. J. Chin. Med. 30 (2-3), 187–194. 10.1142/S0192415X02000442 12230007

[B49] Zaina S. Pettersson L. Ahrén B. Brånén L. Hassan A. B. Lindholm M. (2002). Insulin-like growth factor II plays a central role in atherosclerosis in a mouse model. J. Biol. Chem. 277 (6), 4505–4511. 10.1074/jbc.M108061200 11726660

