# Supplementary material for: Effects of ginseng berry saponins on cardiorespiratory fitness in patients with SCAD: a randomized, double-blinded, placebo-controlled trial
Source: Front Pharmacol. 2026 Jan 22;17:1724232. doi: 10.3389/fphar.2026.1724232 (PMC12872741; doi:10.3389/fphar.2026.1724232)
Supplement: Supplementary file 1 [file Supplementaryfile1.docx]

**Supplementary materials**

| **Inclusion criteria** |
| --- |
| (1) Compliance with western medical diagnostic criteria for SCAD; |
| (2) Angina pectoris CCS classification belongs to grade I-II; |
| (3) The risk stratification of coronary heart disease cardiac rehabilitation belongs to low-risk or intermediate-risk, and cardiopulmonary exercise test can be performed; |
| (4)18 years ≤age≤75 years; |
| (5) LVEF≥40%; |
| (6) Signed informed consent. |
| **Exclusion Criteria** |
| (1) Patients with acute myocardial infarction, unstable angina, within one month after PCI and/or CABG; |
| (2) Patients with absolute andrelative contraindications to cardiopulmonary exercise test; |
| (3) Patients who have taken Zhenyuan capsule in the last 1 month or who have participated in other dinical trials in the last 1 month or who have taken Chinese medicinal preparations (including proprietary Chinese medicines, soups, formulated granules, etc.) in the last 1 week; |
| (4) Combined severe liver disease or liver enzymes (ALT, AST) higher than 3 times the upper limit of normal; severe renal disease or serum creatinine >2.5 mg/dl(male)/2.0 mg/dl (female); |
| (5) Diabetic patients with random blood glucose≥13.7 mmol/L or glycatedhemoglobin≥9.5%; |
| (6) Combined severe cerebrovascular disease, respiratory disease, hematologic disease, malignant tumor and other primary diseases or mental disorders; |
| (7) Pregnant or preparing for pregnancy women, lactating women; |
| (8) Those who are allergic to known components of the study drug. |

**Supplementary Figure 1 Inclusion and exclusion criteria**

**
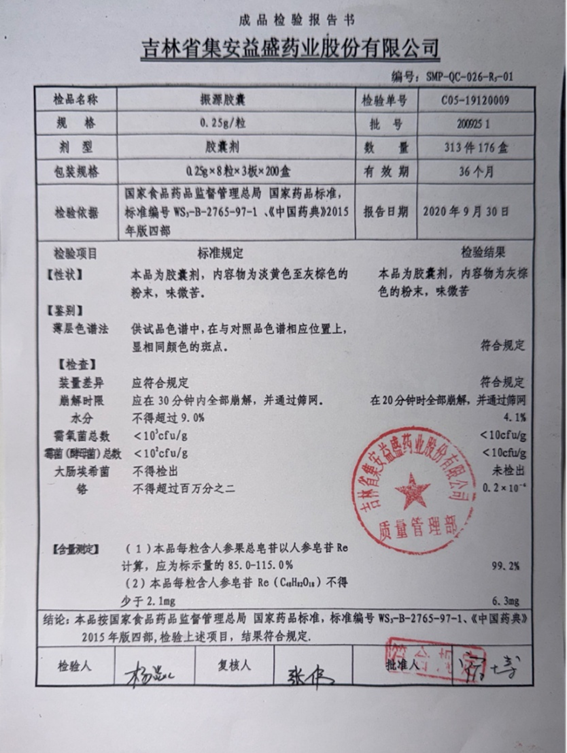
**

**Supplementary Figure 2. Finished Product Inspection Report**


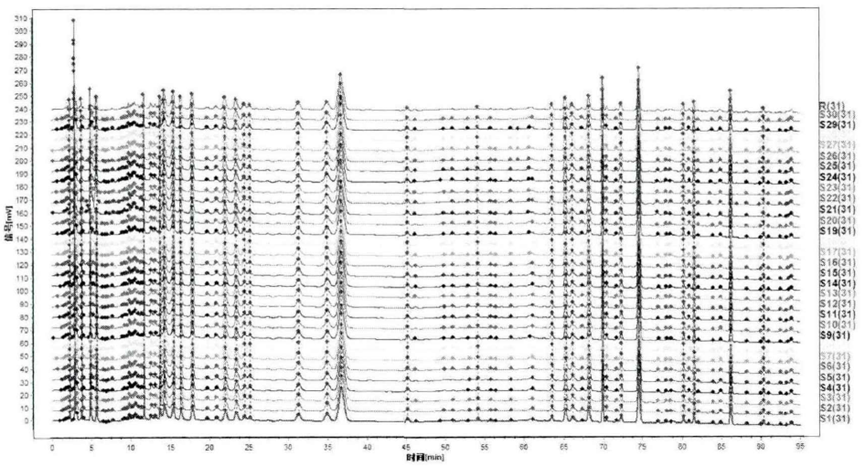


**Supplementary Figure 3.30 HPLC Fingerprint of Batch Zhenyuan Capsules**

**
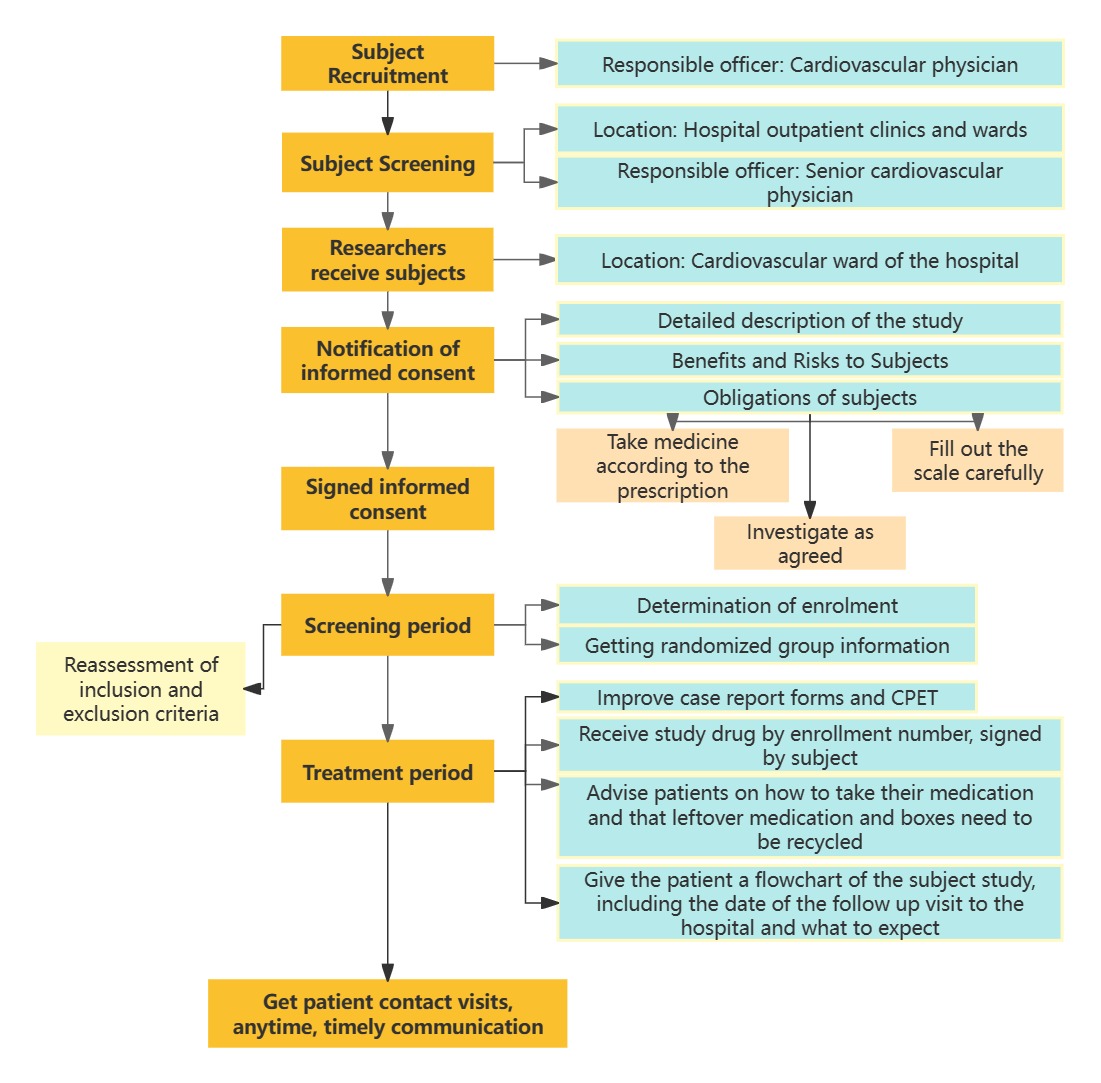
**

**Supplementary Figure 4 Standard operating procedure,SOP**


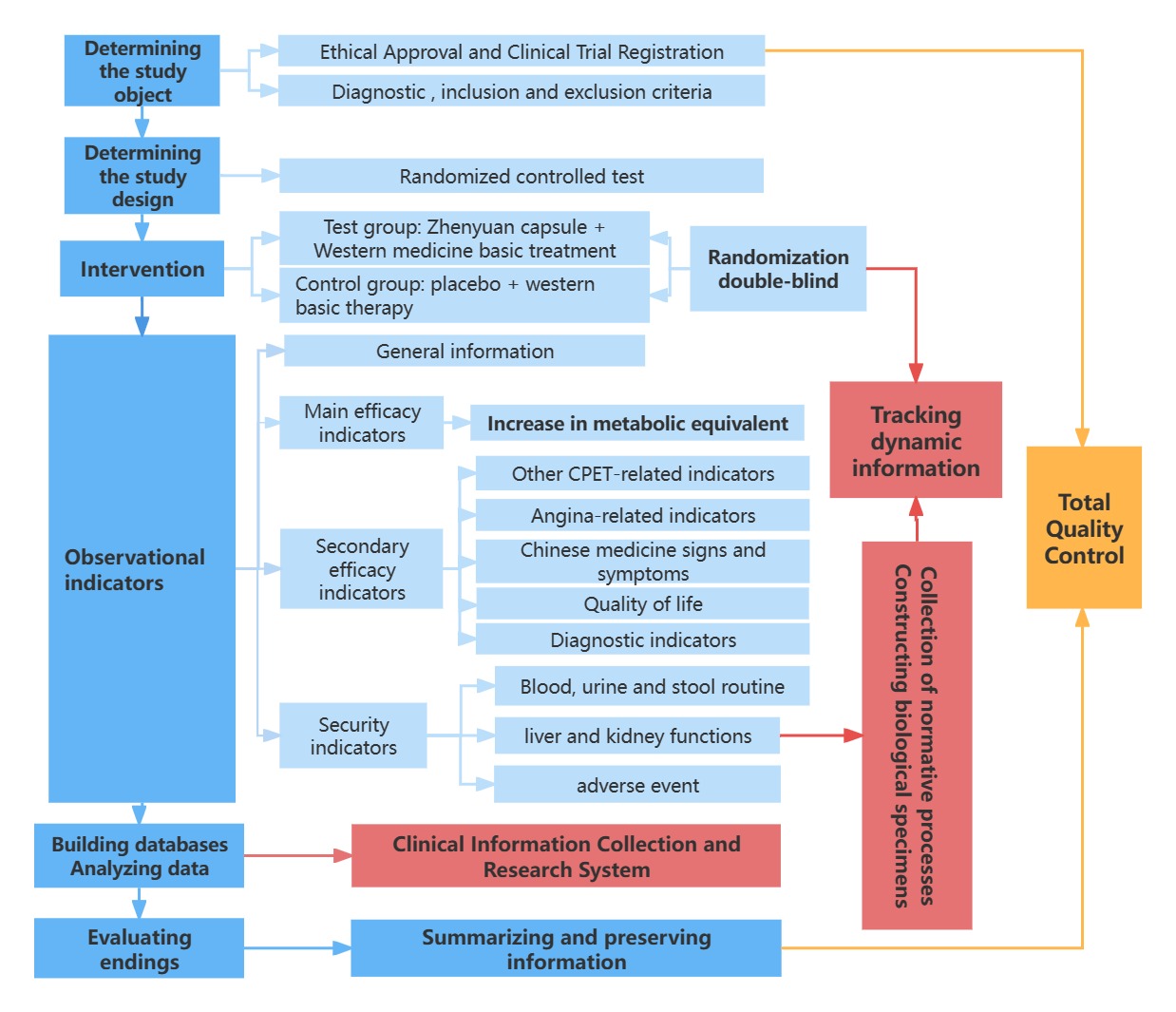


**Supplementary Figure 5 Technological route**

**Supplementary Table 1: Content (%) of Five Saponin Components in 30 Batches of Zhenyuan Capsules**

| **Batch number** | **Re** | **Rc** | **Rb2** | **Rd** | **F2** | **Total** |
| --- | --- | --- | --- | --- | --- | --- |
| S1 | 2.73 | 0.52 | 0.88 | 1.52 | 0.39 | 6.05 |
| S2 | 3.01 | 0.50 | 0.78 | 1.43 | 0.40 | 6.17 |
| S3 | 2.84 | 0.50 | 0.79 | 1.42 | 0.45 | 5.98 |
| S4 | 2.80 | 0.48 | 0.74 | 1.35 | 0.43 | 5.79 |
| S5 | 2.81 | 0.49 | 0.74 | 1.35 | 0.42 | 5.83 |
| S6 | 2.98 | 0.58 | 0.85 | 1.49 | 0.44 | 6.30 |
| S7 | 2.85 | 0.50 | 0.79 | 1.43 | 0.40 | 6.03 |
| S8 | 2.97 | 0.53 | 0.81 | 1.42 | 0.46 | 6.16 |
| S9 | 2.80 | 0.55 | 0.82 | 1.43 | 0.43 | 6.04 |
| S10 | 2.92 | 0.52 | 0.78 | 1.38 | 0.44 | 5.98 |
| S11 | 2.75 | 0.50 | 0.87 | 1.54 | 0.38 | 6.09 |
| S12 | 2.96 | 0.53 | 0.81 | 1.49 | 0.43 | 6.21 |
| S13 | 2.84 | 0.52 | 0.78 | 1.44 | 0.42 | 5.96 |
| S14 | 2.91 | 0.47 | 0.73 | 1.41 | 0.38 | 5.99 |
| S15 | 2.81 | 0.51 | 0.81 | 1.39 | 0.47 | 5.90 |
| S16 | 2.75 | 0.57 | 0.89 | 1.31 | 0.38 | 5.96 |
| S17 | 2.89 | 0.53 | 0.82 | 1.39 | 0.44 | 6.02 |
| S18 | 2.87 | 0.51 | 0.77 | 1.38 | 0.39 | 5.95 |
| S19 | 2.82 | 0.53 | 0.85 | 1.41 | 0.42 | 6.01 |
| S20 | 2.87 | 0.49 | 0.78 | 1.36 | 0.40 | 5.85 |
| S21 | 2.73 | 0.48 | 0.78 | 1.36 | 0.35 | 5.77 |
| S22 | 3.02 | 0.55 | 0.83 | 1.43 | 0.42 | 6.25 |
| S23 | 2.86 | 0.48 | 0.78 | 1.43 | 0.42 | 5.99 |
| S24 | 2.91 | 0.52 | 0.81 | 1.37 | 0.44 | 6.02 |
| S25 | 2.83 | 0.51 | 0.76 | 1.36 | 0.41 | 5.82 |
| S26 | 2.78 | 0.45 | 0.71 | 1.39 | 0.36 | 5.73 |
| S27 | 2.85 | 0.50 | 0.79 | 1.43 | 0.40 | 5.96 |
| S28 | 2.76 | 0.47 | 0.76 | 1.42 | 0.39 | 5.84 |
| S29 | 2.82 | 0.56 | 0.83 | 1.43 | 0.43 | 6.03 |
| S30 | 2.90 | 0.51 | 0.82 | 1.41 | 0.39 | 5.64 |

**Supplementary Table 2 Baseline characteristics of study participants (PPS set)**

| **Variables at baseline** | **All participants（n=92）** | **Test group（n=47）** | **Control group（n=45）** |
| --- | --- | --- | --- |
| **Age(year), mean (*SD*)** | 61.0（8.2） | 62.5（7.8） | 60.0（8.6） |
| **Gender, n (%)** |  |  |  |
| Male | 69（75%） | 32（68%） | 37（82%） |
| Female | 23（25%） | 15（32%） | 8（18%） |
| **Marital Status, n (%)** |  |  |  |
| unmarried | 3（3%） | 1（2%） | 2（4%） |
| married | 32（97%） | 46（98%） | 43（96%） |
| **BMI (kg/m^2^), mean (*SD*)** | 25.3（2.2） | 25.4（2.2） | 25.2（2.3） |
| **Education, n (%)** |  |  |  |
| ＜High school | 17（18%） | 9（19%） | 8（18%） |
| High school | 27（29%） | 14（30%） | 13（29%） |
| ＞High school | 48（53%） | 24（51%） | 24（53%） |
| **SBP (mmHg), mean (*SD*)** | 124（8） | 125（9） | 122（7） |
| **DBP (mmHg), mean (*SD*)** | 76（6） | 77（6） | 74（6） |
| **HR (bpm), mean (*SD*)** | 68（7） | 68（7） | 69（5） |
| **History of hemodynamic reconstruction, n (%)** |  |  |  |
| No | 31（34%） | 17（36%） | 14（31%） |
| PCI | 58（63%） | 28（60%） | 30（67%） |
| CABG | 3（3%） | 2（4%） | 1（2%） |
| **Risk factors, n (%)** |  |  |  |
| Smoking | 53（58%） | 22（47%） | 31（69%） |
| Hypertension | 54（59%） | 28（60%） | 26（58%） |
| Hyperlipidemia | 79（86%） | 44（94%） | 35（78%） |
| Diabetes | 28（30%） | 19（40%） | 9（20%） |
| **TC (mmol/L), mean (*SD*)** | 3.66（0.73） | 3.55（0.73） | 4.02（0.64） |
| **LDL-C (mmol/L), mean (*SD*)** | 2.05（0.66） | 1.99（0.65） | 2.22（0.66） |
| **HDL-C (mmol/L, mean (*SD*)** | 1.11（0.25） | 1.06（0.19） | 1.24（0.35） |
| **TG (mmol/L), mean (*SD*)** | 1.43（0.91） | 1.41（1.03） | 1.56（0.57） |
| **Antiplatelet drug, n (%)** | 84（91%） | 43（92%） | 41（91%） |
| **Lipid-lowering drug, n (%)** | 86（93%） | 45（96%） | 41（91%） |
| **beta-blocker, n (%)** | 50（54%） | 24（51%） | 26（58%） |
| **Nitrate, n (%)** | 17（18%） | 9（19%） | 8（18%） |
| **ACEI/ARB, n (%)** | 35（38%） | 20（43%） | 15（33%） |
| **CCB, n (%)** | 21（23%） | 12（26%） | 9（20%） |
| **Echocardiographic parameters** |  |  |  |
| LVEF，% | 63（63） | 64（64） | 62（61） |
| LVEDD，mm | 46（47） | 46（46） | 47（48） |
| **CPET indicator, mean (*SD*)** |  |  |  |
| Mets at AT (mets) | 3.27（0.79） | 3.24（0.75） | 3.30（0.83） |
| Mets at max (mets | 4.82（0.92） | 4.79（0.95） | 4.86（0.90） |
| VO_2_ peak (ml/min) | 1213（291） | 1188（300） | 1238（283） |
| O_2_ pulse，ml | 10.87（5.84） | 11.67（9.31） | 10.08（2.46） |
| RER | 1.02（0.13） | 1.00（0.14） | 1.04（0.11） |
| HRR1 (bpm) | 17（9） | 16（9） | 19（9） |
| HRR2 (bpm) | 26（10） | 24（10） | 27（11） |
| Duration of exercise (S) | 523（104） | 528（108） | 518（101） |

**Abbreviations:** AT=Anaerobic threshold; Bmi=body mass index; CABG=Coronary artery bypass grafting; DAPT=Duplex antiplatelet therapy; DBP= Diastolic blood pressure; HDL-C=High density lipoprotein cholesterol; HR=Heart rate; HRR1=1min Heart Rate Recovery; HRR2=2min Heart Rate Recovery; LDL-C=Low density lipoprotein cholesterol; LVEDD=Left ventricular end-diastolic diameter; LVEF=Left ventricular ejection fraction; Met=Metabolic equivalent; PCI=Percutaneous coronary intervention; RER=Respiratory exchange rate; SBP=Systolic blood pressure; SCAPT=Single-combination antiplatelet therapy; TC=Total cholesterol; TG=Triglycerides; VO_2_=Oxygen uptake.

**Supplementary Table 3 Effects of Zhenyuan Caspule and placebo on CRF in SCAD (PPS set)**

| **Variables** | **Test group** | | |  |  | **Control group** | | | |  | **Zhenyuan Caspule vs placebo** | |
| --- | --- | --- | --- | --- | --- | --- | --- | --- | --- | --- | --- | --- |
|  | **Baseline value** | **After treatment** | **Mean changes in value (95% CI)** | ***P* value** |  | **Baseline value** | **After treatment** | **Mean changes in value (95% CI)** | ***P* value** |  | **Mean changes in value (95% CI)** | ***P* value** |
| Mets at AT (mets) | 3.24（0.75） | 3.81（0.95） | 0.57（0.31~0.83） | ＜0.001 |  | 3.25（0.84） | 3.40（0.84） | 0.08（-0.24~0.40） | 0.632 |  | 0.49（0.07~0.90） | 0.017 |
| Mets at maximum (mets) | 4.79（0.95） | 5.52（1.15） | 0.73（0.47~0.99） | ＜0.001 |  | 4.86（0.90） | 5.02（0.99） | 0.16（-0.12~0.45） | 0.251 |  | 0.53（0.13~0.94） | 0.004 |
| VO_2_ peak (ml/min) | 1188（300） | 1284（335） | 96（22~169） | 0.010 |  | 1238（283） | 1236（291） | -2（-85~81） | 0.714 |  | 98（-23~218） | 0.249 |
| O_2_ pulse  (ml) | 10.50（2.26） | 11.67（9.31） | 1.17  （-2.02~8.82） | 0.921 |  | 10.02（1.94） | 10.08（2.46） | 0.06  （-1.03~2.06） | 0.797 |  | 1.11  （-2.01~6.76） | 0.870 |
| RER | 1.00（0.14） | 1.09（0.12） | 0.09（0.04~0.13） | ＜0.001 |  | 1.04（0.11） | 1.11（0.11） | 0.07（0.04~0.10） | ＜0.001 |  | 0.02  （-0.04~0.08） | 0.772 |
| HRR1 (bpm) | 16（9） | 21（10） | 5（2~8） | 0.001 |  | 19（9） | 20（13） | 1（-3~5） | 0.432 |  | 4（-1~9） | 0.065 |
| HRR2 (bpm) | 24（10） | 30（12） | 7（3~10） | 0.001 |  | 27（11） | 28（14） | 1（-2~5） | 0.316 |  | 5（0~10） | 0.104 |
| Duration of exercise (S) | 526（136） | 528（108） | 30（-91~88） | 0.516 |  | 535（126） | 518（101） | -23  （-105~63） | 0.286 |  | 53  （-196~151） | 0.223 |

**Abbreviations:** AT=Anaerobic threshold; CRF= cardiorespiratory fitness; HRR1=1min Heart Rate Recovery; HRR2=2min Heart Rate Recovery; RER=Respiratory exchange rate; SCAD= stable coronary artery disease; VO_2_=Oxygen uptake.

| **Group** | | **Before treatment** | **1 month follow-up** | **Difference in value (95% CI)** | **Comparison within group** | |
| --- | --- | --- | --- | --- | --- | --- |
|  |  |  |  |  | **Z Value** | ***P* Value** |
| Test group（n=47） | | 4.79（0.95） | 5.16（1.30） | 0.60(-0.20~1.20) | -2.834 | **0.014** |
| Control group（n=45） | | 4.86（0.90） | 5.06（0.92） | 0.30(-0.05~0.65) | -1.923 | 0.082 |
| **Comparison between groups** | **Statistic** | t=-0.332 | t=0.422 | Z=-1.502 |  | |
|  | ***P* Value** | 0.741 | 0.674 | 0.133 |  | |

Supplementary **Table 4 Metabolic equivalent situation at 1 month follow-up - maximum levels (Mets)**

**Supplementary Table 5 Changes of Mets at maximum across the subgroups.**

|  | Zhenyuan Capsule, n (%) | Placebo, n (%) | Mean (95% CI) | *P* value |
| --- | --- | --- | --- | --- |
| Gender |  |  |  |  |
| Male | 35(70) | 41(82) | -0.56(-0.98~-0.14) | 0.011 |
| Female | 15(30) | 9(18) | -0.46 (-1.51 ~ 0.6) | 0.406 |
| Hypertension |  |  |  |  |
| No | 20(40) | 20(40) | -0.79 (-1.42~-0.16) | 0.019 |
| Yes | 30(60) | 30(60) | -0.22 (-0.78~0.33) | 0.43 |
| Hyperlipidemia |  |  |  |  |
| No | 4(8) | 11(22) | -0.98 (-1.87~-0.1) | 0.05 |
| Yes | 46(92) | 39(78) | -0.36 (-0.83~0.12) | 0.146 |
| Diabetes |  |  |  |  |
| No | 30(60) | 38(76) | -0.52 (-1.03~-0.02) | 0.046 |
| Yes | 20(40) | 12(24) | -0.1 (-0.91~0.71) | 0.813 |

**Supplementary Table 6 Adverse events in the two groups**

| **Group** | **Adverse event** | **Adverse event rate（%）** | **χ^2^** | ***P* Value** |
| --- | --- | --- | --- | --- |
| Test group（n=49） | 1 | 2.04 | 0.000* | 1.000 |
| Control group（n=48） | 1 | 2.08 |  |  |

**Note:** *Corrected χ^2^ value.

**Supplementary Table 7 Analysis of safety indicators before and after treatment in two groups - comparison between groups**

| **indicators** | **before treatment** | | |  | **after treatment** | | |
| --- | --- | --- | --- | --- | --- | --- | --- |
|  | **Test group (n=49)** | **Control group (n=48)** | **Comparison between groups** |  | **Test group (n=49)** | **Control group (n=48)** | **Comparison between groups** |
| WBC(10^9^/L) | 6.20±1.70 | 6.42±1.38 | t=-0.707  *P*=0.481 |  | 6.25±1.60 | 6.38±1.51 | t=-0.530  *P*=0.597 |
| RBC(10^12^/L) | 4.65±0.45 | 4.82±0.36 | t=-2.265  *P*=0.026 |  | 4.65±0.38 | 4.86±0.45 | t=-2.498  *P*=0.014 |
| HGB(g/dL) | 144  (134,154) | 147  (140,156) | Z=-2.074  *P*=0.038 |  | 144  (132,156) | 147  (141,158) | Z=-1.464  *P*=0.143 |
| PLT(10^9^/L) | 217.19±54.65 | 215.35±65.07 | t=0.558  *P*=0.578 |  | 218.17±54.94 | 217.41±61.31 | t=0.057  *P*=0.955 |
| NEUT(%) | 60.88±7.57 | 59.65±8.63 | t=0.678  *P*=0.499 |  | 61.03±7.54 | 59.38±9.21 | t=1.266  *P*=0.209 |
| LYM(%) | 29.59±6.65 | 30.61±8.41 | t=-0.574  *P*=0.567 |  | 29.26±6.25 | 30.97±9.11 | t=-1.240  *P*=0.218 |
| ALT(IU/L) | 19.15(13.95,28.48) | 24.10(16.90,29.00) | Z=-1.396  *P*=0.163 |  | 18.30(11.93,25.93) | 20.80(13.80,32.40) | Z=-1.231  *P*=0.218 |
| AST(IU/L) | 20.35(17.48,23.95) | 21.30(17.40,24.90) | Z=-0.562  *P*=0.574 |  | 20.20(17.03,24.48) | 19.90(17.30,27.10) | Z=-0.090  *P*=0.928 |
| Cr(umol/L) | 73.50(62.00,87.75) | 76.00(67.00,86.00) | Z=-0.745  *P*=0.456 |  | 71.50(60.00,83.75) | 74.00(66.00,84.00) | Z=-0.718  *P*=0.473 |
| BUN(mmol/L) | 14.42(12.11,17.78) | 13.72(12.04,16.68) | Z=-0.771  *P*=0.441 |  | 14.14(12.39,17.08) | 14.56(11.76,17.36) | Z=-0.390  *P*=0.697 |
| Abnormal Urination | 20.93  (9/43) | 23.81  (10/42) | χ^2^=0.101  *P*=0.750 |  | 24.39  (10/41) | 27.03  (10/37) | χ^2^=0.071  *P*=0.790 |
| Abnormal Stool | 0  （0/41） | 0  （0/36） | —— |  | 0  （0/33） | 0  （0/33） | —— |
| Abnormal electrocardiogram | 8.2  （4/49） | 10.4  （5/48） | χ^2^=0.001*  *P*=0.974 |  | 6.1  （3/49） | 6.3  （3/48） | χ^2^=0.000*  *P*=1.000 |

**Note：**Normal data were expressed using mean ± SD, non-normal data were expressed using median (IQR), and count data were expressed using % (number of abnormal cases/total number of cases in the program). *Corrected χ^2^ value.

**Supplementary Table 8 Analysis of safety indicators before and after treatment in two groups - comparison within groups**

| **indicators** | **Test group（n=49）** | | |  | **Control group（n=48）** | | |
| --- | --- | --- | --- | --- | --- | --- | --- |
|  | **before treatment** | **after treatment** | **Comparison within group** |  | **before treatment** | **after treatment** | **Comparison within group** |
| WBC(10^9^/L) | 6.20±1.70 | 6.25±1.60 | t=-0.260  *P*=0.796 |  | 6.42±1.38 | 6.38±1.51 | t=0.238  *P*=0.813 |
| RBC(10^12^/L) | 4.64±0.45 | 4.65±0.38 | Z=-0.102  *P*=0.919 |  | 4.82±0.36 | 4.86±0.45 | Z=-1.078  *P*=0.281 |
| HGB(g/dL) | 144  (134,154) | 144  (132,156) | Z=-0.938  *P*=0.348 |  | 147  (140,156) | 147  (141,158) | Z=-0.955  *P*=0.340 |
| PLT(10^9^/L) | 217.19±54.65 | 218.17±54.94 | Z=-1.006  *P*=0.315 |  | 215.35±65.07 | 217.41±61.31 | Z=-0.135  *P*=0.892 |
| NEUT(%) | 60.88±7.57 | 61.03±7.54 | Z=-0.005  *P*=0.996 |  | 59.65±8.63 | 59.38±9.21 | Z=-0.322  *P*=0.747 |
| LYM(%) | 29.59±6.65 | 29.26±6.25 | Z=0.000  *P*=1.000 |  | 30.61±8.41 | 30.97±9.11 | Z=-0.240  *P*=0.810 |
| ALT(IU/L) | 19.15(13.95,28.48) | 18.30(11.93,25.93) | Z=-0.746  *P*=0.456 |  | 24.10(16.90,29.00) | 20.80(13.80,32.40) | Z=-0.598  *P*=0.550 |
| AST(IU/L) | 20.35(17.48,23.95) | 20.20(17.03,24.48) | Z=-0.783  *P*=0.434 |  | 21.30(17.40,24.90) | 19.90(17.30,27.10) | Z=-0.736  *P*=0.462 |
| Cr(umol/L) | 73.50(62.00,87.75) | 71.50(60.00,83.75) | t=1.703  *P*=0.095 |  | 76.00(67.00,86.00) | 74.00(66.00,84.00) | t=2.630  *P*=0.066 |
| BUN(mmol/L) | 14.42(12.11,17.78) | 14.14(12.39,17.08) | Z=-0.650  *P*=0.516 |  | 13.72(12.04,16.68) | 14.56(11.76,17.36) | Z=-1.103  *P*=0.270 |
| Abnormal Urination | 20.93  (9/43) | 24.39  (10/41) | χ^2^=0.144  *P*=0.705 |  | 23.81  (10/42) | 27.03  (10/37) | χ^2^=0.108  *P*=0.743 |
| Abnormal Stool | 0  （0/42） | 0  （0/33） | —— |  | 0  （0/36） | 0  （0/33） | —— |
| Abnormal electrocardiogram | 8.2  （4/49） | 6.1  （3/49） | χ^2^=0.000*  *P*=1.000 |  | 10.4  （5/48） | 6.3  （3/48） | χ^2^=0.136*  *P*=0.712 |

**Note：**Normal data were expressed using mean ± SD, non-normal data were expressed using median (IQR), and count data were expressed using % (number of abnormal cases/total number of cases in the program). *Corrected χ^2^ value.

**Cardiopulmonary exercise test (CPET)**

**1. Exercise Load Protocol:**

We have clearly outlined the specific protocol used to calculate metabolic equivalents (METs) and peak oxygen uptake (VO₂ max): The exercise cardiopulmonary testing system employed in this study is the MasterScreen CPX from JAEGER, Germany. Symptom-limited maximal CPET was conducted according to the standards of the CPET laboratory at the University of California, Los Angeles Medical Center. Prior to each examination, the system undergoes separate calibration for airflow, O₂, and CO₂ measurements, followed by calibration using a standard metabolic simulator before proceeding with CPET.

The basic procedure is as follows: First, the subject completes a full set of static pulmonary function tests in a seated position, including measurement of vital capacity, total lung capacity, maximum minute ventilation, pulmonary diffusion capacity, and other pulmonary function indicators. The subject then performs the exercise protocol on a stationary bicycle, beginning with a 3-minute rest period; followed by a 3-minute warm-up at 55-65 rpm without exercise load; Subsequently, the subject pedals at the exercise power estimated by the system until exercise termination due to symptom limitation. Finally, the subject recovers on the bicycle for 4-10 minutes. All parameters throughout the seated and cycling phases are recorded, including total duration, dynamic electrocardiogram, blood oxygen saturation, blood pressure, exercise power, and various pulmonary ventilation and gas exchange metrics.

**2. Objective Criteria for Test Termination:**

Beyond the subjective criterion of “symptom limitation,” we have explicitly defined objective indicators requiring immediate test termination. All tests strictly adhere to the following standards:

1. Resting heart rate ≥ 120 beats per minute;
2. Resting respiratory rate ≥ 30 breaths per minute;
3. Oxygen saturation ≤ 90%; Pre-exercise systolic blood pressure (SBP) ≥180 mmHg or diastolic blood pressure (DBP) ≥110 mmHg;
4. Weight fluctuation exceeding ±1.8 kg within 72 hours;
5. New ischemic changes clearly observable on resting electrocardiogram (ECG);
6. Unstable angina pectoris;
7. Confirmed or suspected pseudoaneurysm;
8. Preoperative arterial dissection; Infectious shock and sepsis; Preoperative for severe valvular disease or acute heart failure in cardiomyopathy; Parkinson's disease, Parkinsonian syndromes; Tremor, restless legs syndrome, and other neurological, motor system disorders, or rheumatic diseases where exercise is deemed likely to exacerbate symptoms by the clinician;
9. Severe stenotic valvular disease; concurrent acute bronchial asthma or chronic obstructive pulmonary disease, pulmonary edema; bradycardia (resting heart rate <50 bpm), tachycardia, or high-degree atrioventricular block; left main coronary artery stenosis; electrolyte abnormalities; patient inability or unwillingness to cooperate.

**3. Training and Operational Consistency Assurance for Test Personnel:**

We have supplemented the quality control measures implemented to ensure data reliability:

1. Personnel Training: All CPET operators have undergone standardized training and passed assessments.
2. Equipment Calibration: Gas analyzers and flow meters are calibrated per manufacturer specifications before each test; bicycle dynamometer loads undergo periodic calibration.
3. SOP Compliance: The entire testing process—from subject preparation, equipment fitting, resting data collection, exercise testing, to recovery monitoring—strictly follows documented Standard Operating Procedures.

**Serum Proteomics Analysis**

**1 Major Instruments and Reagents**

**1.1 Major Instruments**

Vortex Shaker (Shanghai Qite Analytical Instruments, Model: XW-80A)

Centrifuge (Eppendorf, Model: 5430R)

Microplate Reader (Thermo, Model: Multiskan SkyHigh)

Electrophoresis System (GE Healthcare EPS601)

Constant Temperature Incubator (Shanghai Pudong Rongfeng Scientific Instrument Co., Ltd., Model: HH.S4)

High Performance Liquid Chromatograph (Thermo Scientific EASY-nLC 1000 System (Nano HPLC))

Mass Spectrometry System (Thermo, Model: Q-Exactive)

**1.2 Main Reagents**

Urea (161-0731, Bio-Rad)

Acetone (179124, Sigma-Aldrich)

Bromophenol Blue (A602230-0025, Sangon)

Glycerol (A100854-0100, Sangon)

DTT (D9163-5G, Sigma-Aldrich)

SDS (161-0302, Bio-Rad)

Tris (A6141, Sigma)

HCl (10011018, Sinopharm)

NH₄HCO₃ (A6141, Sigma)

Trypsin (HLS TRY001C PN: 020201308)

1× Phosphate-buffered saline (PBS)

ProteoExtract™ High Abundance Protein Removal Kit (122640, Sigma-Aldrich)

Buffer Preparation

1. SDT Lysis Buffer: 4% SDS, 100 mM Tris-HCl, pH 7.6

② UA Buffer: 8M Urea, 150mM Tris-HCl, pH 8.0

③ 5X Loading Buffer: 10% SDS, 0.5% Bromophenol Blue, 50% Glycerol, 500mM DTT, 250mM Tris-HCl, pH 6.8

Coomassie Brilliant Blue R-250 (A610037-0025, Sangon)

SDS-PAGE Precast Gel (M00657, Genscript)

SDS Electrophoresis Buffer Kit (M00138, Genscript)

BCA Protein Concentration Assay Kit (P0012, Beyotime)

DTT (Sigma-Aldrich, D9163-5G)

IAA (Sigma-Aldrich, V900335-25G)

Trypsin buffer (4μL Trypsin in 40μL NH₄HCO₃)

Trypsin (HLS TRY001C, PN: 020201308)

Mobile Phase A: 100% ultrapure water, 0.1% formic acid

Mobile Phase B: 84% acetonitrile, 0.1% formic acid

Formic acid: (FA, 06450 Fluka)

Methanol: (Sigma, 34851)

Column: Thermo Scientific Acclaim PepMap100, 100μm*2cm, nanoViper C18

Analytical column: Thermo Scientific EASY column, 10cm, ID75μm, 3μm, C18-A2

**2 Primary Methods**

**2.1 Sample Sources**

Serum samples were collected from all patients enrolled in the second randomized controlled trial before treatment and 3 months post-treatment. Following data analysis, 8 patients exhibiting significantly improved METs scores after 3 months of Zhenyuan Capsule administration were selected.

**2.2 Serum Sample Collection**

Blood samples were collected by professional nursing staff in the Cardiovascular Department III ward of Xiyuan Hospital, China Academy of Chinese Medical Sciences. Fasting blood was drawn from the antecubital vein in the morning. The first draw occurred before treatment initiation, and the second draw was performed 3 months after medication administration. Samples were allowed to clot naturally at room temperature for 15 minutes and centrifuged within 30 minutes. Centrifuge parameters were set to: temperature 4℃, time 15 minutes, speed 3000 rpm. Following centrifugation, serum was transferred to 1.5 ml cryovials using pipettes. Each cryovial was clearly labeled with the patient's name, gender, enrollment number, draw sequence number, and draw date. Finally, cryovials were stored in a -80℃ freezer.

**2.3 Serum Sample Grouping**

Clinically collected serum samples were divided into two groups: pre-treatment with the VibroSource capsule (Group A) and 3 months post-treatment (Group B), with 8 cases in each group. Proteomics research and analysis were then conducted.

**2.4 Serum Proteomics Study**

**2.4.1 Removal of High-Abundance Proteins**

(1) Sample Preparation: Remove stored serum samples from the -80℃ freezer. Thaw gently by gently shaking on ice. Dilute 60 μL of serum with 10× binding buffer (600 μL) in a clearly labeled separation tube.

(2) Column pretreatment: Remove the blue cap from the column and invert it onto a paper towel to drain residual buffer; detach the bottom connector, locate a suitable buffer collection tube, and insert the column; add 0.85 mL binding buffer to the column, allowing it to flow through the resin bed by gravity. Discard the used buffer collection tube and place the column into a new collection tube.

(3) Removal of Albumin/IgG: Add the prepared diluted sample to the column and allow it to flow through the resin bed. Collect the mobile phase, then wash the column with 600 μL of binding buffer. Allow the binding buffer to flow through the resin bed and collect the eluate. Repeat the above procedure to collect additional eluate.

**2.4.2 Serum Protein Sample Preparation**

Serum proteins were extracted using the acetone precipitation method. High-abundance serum samples were removed by adding 800 μL of pre-chilled acetone to each 200 μL sample. Incubate at -20℃ for 4 hours, Centrifuge at 4℃, 12000g for 30 min. Discard the supernatant. Wash the pellet 2–3 times with pre-chilled acetone. Allow to stand at room temperature for 10 min to evaporate residual acetone, leaving the protein pellet. Resuspend the pellet in SDT lysis buffer. Determine protein concentration using the BCA method. Finally, assess protein sample extraction by SDS-PAGE electrophoresis.

**2.4.3 Protein Quantification (BCA Method)**

Determine serum sample concentration after high-abundance protein removal using the bicinchoninic acid (BCA) method per protocol:

(1) Prepare BCA working solution according to kit instructions: Based on the volume of BCA protein standard and sample to be tested, prepare working solution at a ratio of 50 volumes of BCA reagent to 1 volume of Cu reagent. Mix thoroughly. Add 200 μl of working solution to each well of a 96-well plate.

(2) Dilute the BCA protein standard to a concentration of 5 mg/mL. Add the diluted protein standard to the 96-well plate in volumes of 0, 2, 4, 6, 8, 12, 16, and 20 μL. For wells containing less than 20 μL, top up with sterile double-distilled water;

(3) Add 20 μL of the test sample to the 96-well plate;

(4) Add 200 μl of prepared BCA working solution to the 96-well plate containing protein standards and samples;

(5) Incubate the plate at 37℃ for 30 minutes, then allow it to cool to room temperature;

(6) Measure the absorbance at 562 nm using a microplate reader and calculate the sample concentration based on the standard curve.

**2.4.4 SDS-PAGE Protein Electrophoresis**

Perform SDS-PAGE electrophoresis on 20 μl of serum sample from each case. Stain with Coomassie Brilliant Blue, decolorize until the background is clear. Detailed steps are as follows: ① Gel Preparation: Clean the glass plates, combs, and slides, then air-dry them. Align one end of the glass plate with the slide, clamp them together with forceps, and secure them on the gel casting stand. Prepare equal volumes of separating gel and concentrating gel according to the instructions. Add the separating gel first, followed by the concentrating gel, and allow the gel to solidify. ② Sample loading and electrophoresis: Remove the comb. Add standard protein markers and test samples to the gel wells respectively. Fill the electrophoresis chamber with prepared running buffer. Apply 90V for 30 minutes, then increase to 120V until the bromophenol blue indicator reaches the bottom of the gel. ③ Stop electrophoresis, carefully remove the gel, and stain with Malachite Green for 1 hour. After 1 hour, rinse the gel three times with deionized water, then decolorize in decolorizing solution for 2 hours until protein bands are clearly visible and the gel background is thoroughly decolorized. Finally, rinse the gel again, scan, and save the gel image.

**2.4.5 Protein Digestion**

Add DTT (40 mM) to each sample and stir at 600 rpm for 1.5 h (37℃). After cooling to room temperature, add 20 mM IAA to the mixture to block reducible cysteine residues and incubate in the dark for 30 min. Transfer samples to separate filters. Wash three times with 100 μl UA buffer, followed by two washes with 100 μl 25 mM NH₄HCO₃ buffer. Finally, add trypsin (trypsin:protein (wt/wt) 1:50) to the sample and incubate at 37℃ for 15-18 hours (overnight). Collect the resulting peptides as the filtrate. Each peptide sample was desalted on a C18 column (Empore™ SPE Cartridges C18 (standard density), bed I.D. 7 mm, volume 3 ml, Sigma), concentrated by vacuum centrifugation, redissolved in 40 µl of 0.1% (v/v) formic acid, centrifuged at 12000g for 10 min, and the supernatant was collected for injection and analysis.

**2.4.6 Protein Analysis Using Nanoliter-Scale Reverse-Phase Chromatography with Q Exactive**

Protease-digested samples were separated using the Easy nLC nanoliter-flow HPLC system. A complex solution was first prepared. Subsequently, 20 μl of this complex solution was combined with the fraction obtained from reverse-phase separation. The mixture was centrifuged at 12000g for 10 minutes. After centrifugation, the supernatant was aspirated and loaded onto the loading column using the sandwich method (10 μl). The column was equilibrated with 95% mobile phase A. Samples were loaded onto the pre-column via the autosampler and separated on the analytical column at a loading pump flow rate of 300 nl/min for 15 minutes. The separation flow rate was 300 nl/min, with the following gradient:

| Time（minutes） | Mobile Phase B Ratio（%） |
| --- | --- |
| 0 | 5 |
| 8 | 15 |
| 40 | 25 |
| 65 | 35 |
| 70 | 95 |
| 85 | 4 |
| 90 | 5 |

Serum samples were chromatographically separated and analyzed by mass spectrometry using a Q Exactive mass spectrometer. The detection mode was positive ion, with a parent ion scan range of 300–1800 m/z. The primary mass spectrometer resolution was 70000 at 200 m/z, with an automatic gain control target of 1e6, maximum ion time of 50 ms, and dynamic exclusion time of 60 s. Mass-to-charge ratios for peptides and peptide fragments were acquired as follows: Twenty fragment spectra (MS2 scans) were collected after each full scan. MS2 activation type was HCD, with an isolation window of 2 m/z, secondary mass spectrometry resolution of 17,500 at 200 m/z, normalized collision energy of 30 eV, and a base fill level of 0.1%.

**2.4.7 Mass Spectrometry Data Processing**

The raw data format for mass spectrometry analysis is RAW. Database checking, identification, and quantitative analysis were performed using MaxQuant software (version 1.5.3.17) with the following parameters:

| Items | Value |
| --- | --- |
| Enzyme | Trypsin |
| Max Missed Cleavages | 2 |
| Fixed modifications | Carbamidomethyl (C) |
| Variable modifications | Oxidation (M) |
| Main search | 6 ppm |
| First search | 20ppm |
| MS/MS Tolerance | 20ppm |
| Database pattern | Reverse |
| Include contaminants | True |
| protein FDR | ≤0.01 |
| Peptide FDR | ≤0.01 |
| Peptides used for protein quantification | Use razor and unique peptides |
| Time window (match between runs) | 2min |
| protein quantification | LFQ |
| min. ratio count | 1 |

**2.4.8 Bioinformatics Analysis**

**(1) GO Functional Annotation and KEGG Pathway Annotation**

Blast2GO was employed to annotate the target protein set with GO terms, describing the attributes of target genes and gene products across three domains: Cellular Component (C), Molecular Function (F), and Biological Process (B). KEGG pathway annotation was performed on the target protein set using KAAS (KEGG Automatic Annotation Server) software to obtain information on the gene pathways involving the target protein sequences.

**(2) Enrichment Analysis**

Fisher's exact test was employed to conduct enrichment analysis of GO annotations or KEGG pathway annotations for the target protein set.

**(3) Protein-Protein Interaction (PPI) Network Analysis**

Direct and indirect interactions among target proteins were identified using information from the STRING (http://string-db.org/) database. CytoScape software (version 3.2.1) was employed to generate interaction networks and perform network analysis.

**3 Statistical Analysis**

Statistical analysis was performed using SPSS 26.0 software. Quantitative data are expressed as mean ± standard deviation, while categorical data are presented as counts and percentages. For quantitative data, t-tests were applied when data followed a normal distribution with equal variances; otherwise, nonparametric tests were used. Categorical data were analyzed using chi-square tests. A *P* value ＜0.05 was considered statistically significant.
